# Integrating EEG Sensors with Virtual Reality to Support Students with ADHD

**DOI:** 10.3390/s26031017

**Published:** 2026-02-04

**Authors:** Juriaan Wolfers, William Hurst, Caspar Krampe

**Affiliations:** 1 Information Technology Group, Wageningen University and Research, Building No. 201 (Leeuwenborch), Hollandseweg 1, 6706 KN Wageningen, The Netherlands; juriaan.wolfers@wur.nl; 2 Marketing and Consumer Behaviour Group, Wageningen University and Research, Hollandseweg 1, 6706 KN Wageningen, The Netherlands; caspar.krampe@wur.nl

**Keywords:** virtual reality, Brain–Computer Interaction, ADHD, education, electroencephalogram

## Abstract

Students with attention deficit hyperactivity disorder (ADHD) face a continuous challenge with their attention span, putting them at a greater risk of academic or psychological difficulties compared to their peers. Innovative communication technologies are demonstrating potential to address these attention-span concerns. Virtual Reality (VR) is one such example, and has the potential to address attention-span difficulties among ADHD students. Accordingly, this study presents an EEG-based multimodal sensing pipeline as a methodological contribution, focusing on sensor-based data acquisition, signal processing, and neurophysiological interpretation to assess attention in VR-based environments, simulating a university supply chain educational topic. Thus, in this paper, a sequential exploratory approach investigated how 35 participants experienced an interactive VR-learning-driven supply chain game. A Brain–Computer Interaction (BCI) sensor generated insights by quantitatively analysing electroencephalogram (EEG) data that were processed through the proposed pipeline and integrated with subjective measures to validate participant’s subjective feelings. These insights originated from questions during the experiment that followed the Spatial Presence and Technology Acceptance Model to form a multimodal assessment framework. Findings demonstrated that the experimental group experienced a higher improved attention, concentration, engagement, and focus levels compared to the control group. BCI results from the experimental group showed more dominant voltage potentials in the right frontal and prefrontal cortex of the brain in areas responsible for attention, memory, and decision-making. A high acceptance of the VR technology among neurodiverse students highlights the added benefits of multimodal learning assessment methods in an educational setting.

## 1. Introduction

Advances in consumer-level sensor-driven technologies, particularly for electroencephalogram (EEG), offer opportunities to create adaptive, personalised learning environments that respond to the attention and cognitive state of neurodiverse students; a population that often faces significant challenges in sustaining focus and engagement. Attention deficit hyperactivity disorder (ADHD) is a common neuropsychiatric syndrome that is characterised by inattention, hyperactivity, impulsive acting, and hasty decision-making [1]. ADHD develops during childhood and often persists into adulthood, with potential serious consequences on education if it is not properly identified and catered for [2]. Examples of primary treatments involve medication, coaching, and strategy instructions [3]. Other feasible interventions are the use of mindfulness meditation and background music to improve behavioural and neurocognitive impairments [4,5]. Existing studies into the cognitive cause of ADHD reveals an executive function impairment in the prefrontal cortex of the brain [6], the region responsible for executive functions, such as planning and decision-making [2,4,5,6,7]. ADHD is estimated to affect about 5–8% of children (depending on the geographical region and assessment criteria) and 2.5% of adults worldwide [8]. Thus, there is scope for developing alternative assessments, practice, and a deeper understanding of ADHD within educational environments. Particularly, students at all levels with ADHD face a continuous challenge with their attention span, putting them at a greater risk of academic or psychological difficulties compared to their peers [3].

Currently, there is a limited volume of practical implementations of multimodal sensing pipelines, combining sensor-based data acquisition with Virtual Reality (VR)-based learning for university students with ADHD. To-date, state-of-the-art research has predominantly focused on VR as an assessment or behavioural training tool for children with ADHD, with comparatively little emphasis on immersive, EEG-informed VR learning environments tailored to adults in higher education. Yet, VR can be a promising tool to address existing challenges, offering unique opportunities to enhance insights into managing ADHD in adulthood. For example, Cuber et al. [9] investigated the technology’s efficacy for homework, along-side automated feedback indicting increased motivation and efficiency when using VR.

Our work incrementally extends prior studies through the development of a dedicated VR application and test case in the context of supply chain education, while also exploring the integration of EEG with interactive educational VR for adults with ADHD. Central to this, is an EEG-based multimodal sensing framework for post hoc analysis, and with the potential to support system-level adaptation (e.g., monitoring of attention-related neural activity) and future personalisation of interaction, feedback, or task difficulty within VR-based learning environments. Thus, this study aims to investigate the use of VR to facilitate attention-span improvements particularly within higher education, with findings intended to support the aforementioned knowledge gap. By integrating sensor-driven (i.e., EEG-based) insights with interactive VR, the system not only measures outcomes but also creates a framework for adaptive, closed-loop educational experiences that respond to neurodiverse student needs.

Specifically, this study is centred on the following research questions, RQ1) How can a VR application be designed to make an intangible supply chain education topic tangible? Also, RQ2) How do students perceive the supply chain education environment in terms of the Presence and Technology Acceptance questionnaire model? Addressing these questions will be achieved by providing quantitative EEG results complemented with a pre- and post-survey process. While the present study focuses on offline analysis, the proposed sensing architecture establishes a foundation for future real-time and personalised VR learning systems, with the findings from this study having the potential to enhance, strengthen, and future-proof the VR deployment in the educational sector.

The remainder of this paper is as follows. Section 2 provides a background discussion on related works on ADHD and VR, with Section 3 outlining the methodology for our study. Results are presented in Section 4, and the paper is concluded in Section 5.

## 2. Background

To date, existing research on the use of VR to support attention, learning, and cognitive functioning in educational contexts has been investigated primarily at an earlier stage in the educational journey, such as in primary and secondary schools. Thus, in this section, the discussion highlights the potential of VR for individuals with ADHD and the current gaps in knowledge that motivate further investigation.

### 2.1. VR for Education Support

Previous work into the use of VR on students with ADHD has focused predominantly on children. For example, a study into the effect of distractors on sustained attention and hyperactivity in youth by Hong et al. has shown that VR is a useful tool for investigating the effect of distractors on individuals [5]. A study by Areces et al. on the use of VR for the diagnosis of ADHD has shown that the AULA Nesplora VR test can be used to establish a differential diagnosis of ADHD presentations in children under different contextual conditions [6]. A meta-analysis by Romero-Ayuso et al. on the effective use of VR interventions for children and adolescents with ADHD concluded an improvement in sustained attention, attentional vigilance measures, and improvements in correct responses [10].

Studies into the learning capabilities of VR on pupils without ADHD indicates mixed results. Specifically, a study by Makransky et al. found that VR increases students’ understanding, especially when they are engaged or stimulated to use the technology [11]. However, understanding how to effectively design the application to harness the affective appeal of virtual environments, while aiming to improve and enhance the learning of students, is a fundamental challenge. Initial situational interest can be a first step in promoting learning, but not learning better compared to conventional methods per se [11]. Extraneous overload by the students, utilisation of the hedonic system instead of the utilitarian system, and ‘bells and whistles’ within the virtual environment leads to less active learning and focuses more on the entertainment element of the technology [11]. For example, studies conducted by Ip et al. and Rus-Calafell et al. have examined the use of VR technology to enhance social skills in children with autism spectrum disorder and schizophrenia. These studies, along with a study by Xiaozhe et al. on creativity, have demonstrated that the technology resulted in significant improvements in social functioning, communication, expression, and immersion [7,12,13]. Additionally, creativity has been found to increase at an individual level, while maintaining a more stable focus or attention.

As students with ADHD tend to labour with their attention span continuously, and this generally leads to more difficulties with academic and social functioning in an educational college environment [3]. VR has the potential to stimulate learning and attention-related challenges. The technology’s highly immersive, customisable, and safe virtual environment indicators are strong aspects of the technology for this purpose. Nevertheless, existing research into the usage of VR for ADHD, have been primary focused on children and adolescents between the age of 7 and 17, often only addressing the usage of the technology to diagnose ADHD. Relatively less is known about the effect of attention-span- and learning-related capabilities of VR in adult learners with ADHD. Moreover, only a few studies integrate multi-sensor setups—including a VR with a Brain–Computer Interaction (BCI) sensor—to complement qualitative findings with quantitative EEG measures of attention span in adult learners in higher education with VR. Additionally, there is a lack of research and guidelines on practical implementations of VR design for educational purposes. Hence, the objective of this research is to investigate the potential of enhancing attention-span improvements in VR for ADHD students, demonstrating a novel approach of VR implementation and sensor-based measurements in a higher educational setting for students with ADHD.

### 2.2. EEG Sensors Coupled with VR

To achieve this, a VR learning environment focused on the supply chain domain will be developed. Following a sequential exploratory design with a control group, the virtual role-play environment will be assessed using a pre- and post-assessment via surveys, while EEG data are recording during the experiment. Unlike prior ADHD VR studies, which focuses on children and adolescents, this study targets university students and integrates multiple sensors—including a VR HMD and a BCI EEG headset—to investigate the learning capability of VR for ADHD students in a university setting while quantitatively assessing neurophysiological effects. To date, there is an existing body of work on the use of EEG for educational applications. For example, EEG has been adopted as a non-invasive method for measuring neural correlates of attention, cognitive workload, and engagement in learning contexts. Existing studies have demonstrated that EEG-derived indicators (e.g., alpha, beta, and theta band activity) are associated with attentional processes, mental effort, and executive functioning [14]. In educational research, EEG has been used to objectively assess learners’ cognitive states beyond self-reported measures, which are often subjective and prone to bias [15].

Furthermore, EEG has been considered in cognitive and educational research to investigate attentional processes and workload during learning tasks. Whereby, variations in EEG frequency bands, such as the theta, alpha, and beta activity, reflects changes in sustained attention and executive control. EEG has also been employed to measure cognitive load and learning-related mental effort, offering insights that are more challenging to capture through self-reported measures alone [16]. Such physiological measures are particularly valuable when evaluating immersive learning technologies, where learners may struggle to accurately articulate their cognitive experiences. The use of EEG in VR-based learning environments has gained increasing attention due to the potential to induce both heightened engagement and increased cognitive demands. Furthermore, Parong et al. similarly outlined that VR environments can lead to poorer learning outcomes compared to less immersive media when cognitive load exceeds learners’ processing capacity [17]. These findings highlight the importance of monitoring learners’ cognitive states during VR-based instruction, for which EEG provides a valuable objective indicator.

Beyond cognitive load assessment, EEG has also been used to investigate attentional regulation and mental states in adaptive and neuro-ergonomic systems. For instance, passive BCI approaches use EEG signals to infer users’ cognitive or attentional states without requiring intentional control [18]. While much of this work has focused on operational or human–machine interaction contexts, it demonstrates the feasibility of using EEG to continuously assess attention in real-time environments.

In relation to ADHD, EEG has long been used to examine atypical neural activity associated with attentional deficits. Elevated theta activity and altered theta–beta ratios have frequently been reported in individuals with ADHD, reflecting challenges in sustained attention and executive control [19]. Although EEG has been widely applied in clinical and diagnostic research on ADHD, its integration into immersive educational environments remains limited. Most existing studies focus on children or clinical assessment contexts rather than learning-oriented VR applications for adult students. Despite its potential, the combined use of EEG and VR in educational research presents methodological challenges [20]. EEG data collection in immersive environments may be affected by movement artifacts and signal noise, and there remains a lack of established guidelines on how EEG metrics should be interpreted to inform educational VR design. Furthermore, research specifically targeting higher-education learners with ADHD is scarce, particularly in domains requiring sustained attention and complex cognitive processing, such as supply chain education.

## 3. Methodology

In this section, we present the design of the aforementioned application, methodology processes for the pre/post-survey, and the integration of the EEG sensors for the quantitative data collection.

### 3.1. VR Application Overview

When using HMD-based VR, the user is surrounded by a given three-dimensional environment that enhances their perception of the virtual world, elevating their sense of presence and allowing the individual to interact and potentially learn from the given scenario displayed in the headset [21]. Thus, VR offers a different approach to learning compared to reading or a video, as in a VR application, the context can be shaped by the creator in such a way that it follows a clear path that engages and stimulates the user to learn from its experience.

#### 3.1.1. Application Design

The main objective of the VR experience created for this study was to mimic an educational topic taught at the Wageningen University, specifically learning the fundamentals of Supple Chain Management (SCM), which is part of the supply chain educational curriculum. SCM was chosen as it represents an intangible topic that can be difficult to grasp for (more visual-oriented) students, levering the visual and novel learning advantages of VR. Furthermore, SCM is a particularly relevant test case for ADHD-focused learning research because it requires sustained attention, working memory, dynamic decision-making under uncertainty, and the integration of multiple information streams. In addition, two environments (a typical Wageningen University classroom and a calm nature environment) were created to explore the VR setting on attention span and to leverage the customisability of VR. The main rationale behind the supply chain game was to teach the participant about the difficulty of Inventory Management and the importance of transparency and communication in supply chain networks.

**Figure 1:**
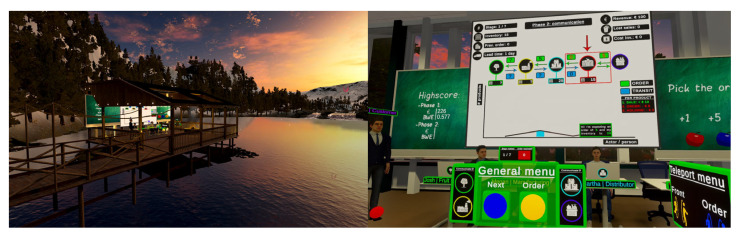
(**Left**) Nature environment and (**Right**) NPC location in the classroom environment.

A 3-min virtual lecture by the research team was displayed to all participants in the VR environment, explaining the fundamentals of SCM and practicalities of interaction with the game. Within the game, the player must decide how many products to order to satisfy their customer’s demand while maximising profit and minimising lost sales and inventory costs. The player takes on the role as an inventory manager for a grocery store and has to manage the inventory of apples. The game is then played over two phases, representing a gameplay feature: (1) a no-communication phase, showcasing the player the importance of information for optimal decision-making by letting them play the game without external information; and (2) a communication phase, allowing the player to communicate with the other Non-Player Character (NPC) actors (Figure 1: Right) to reveal information regarding inventory and demand.

The distributor and the customer were the player’s main actors for interactivity, responsible, respectively, for ordering products and satisfying demand. A processor and a fruit farmer were the other two actors further up the chain. Each actor has their own inventory and logic behind their ordering amount, with the exclusion of the customer.

**Figure 2:**
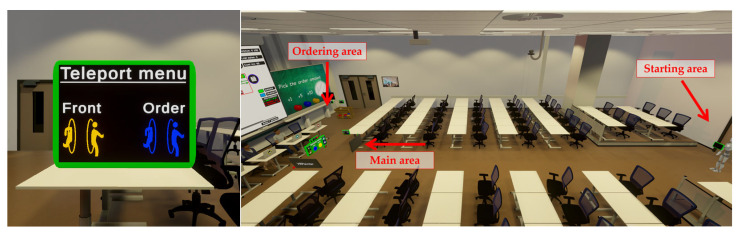
(**Left**) Teleport menu and (**Right**) environment overview.

To enhance experiment repeatability and evaluate the participants’ ability to handle unexpected situations, a predetermined demand per stage is implemented via the customer actor. Examples include running out of inventory halfway through the week due to a big increase in demand, and experiencing a bullwhip effect (amplified variability across the SC). The customer would place an order in these 7 simulated days with 5, 8, 12, 8, 8, 5, 3 products, respectively, starting with an inventory of 10 on day 1. The second communication phase also follows the same ordering quantity, but communication with the actors reveals actor-specific inventory and expected order quantities. Figure 2 displays an overview of the environment and a teleport menu that was introduced (one at the start, one at the main area and one at the ordering area) to allow the user to teleport through the environment rather than walking to avoid motion-sickness related symptoms.

**Figure 3:**
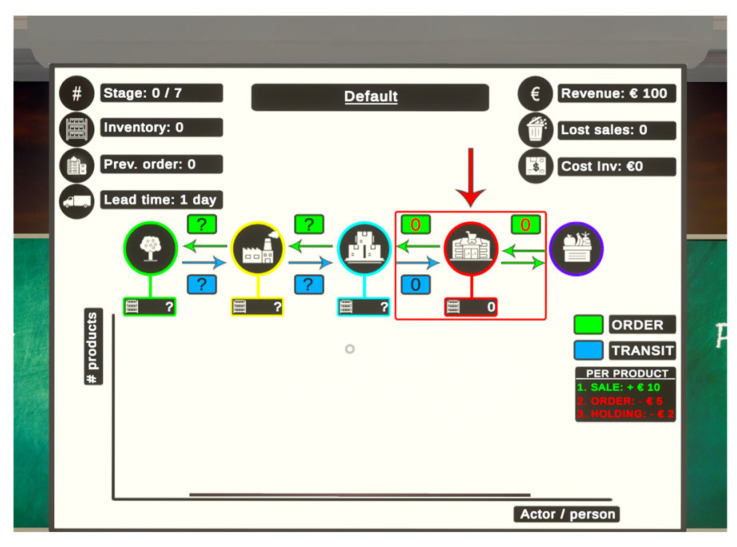
User Interface design schematic.

NPCs were added to ensure a repeatable experience per participant, whilst allowing for different outcomes based on the actions of the player. The fruit supplier and the customer only served as a placeholder to provide ‘inventory and demand’ information to the player, respectively. A beneficial design principle adopted during the development of the supply chain game was to facilitate a general interface that provides all the important information needed to play the supply chain game. This menu should be intuitive and easy to understand for novice users. Figure 3 shows the final design of the main menu present in the game, displaying necessary information needed for decision-making, such as inventory and incoming order amounts. It should be noted that there are more ways of displaying these elements in-game. However, the setting of the supply chain game was played in a classroom, following a conventional setting, simulating a reality that often uses a projector screen to display a presentation (e.g., PowerPoint) during a lecture. Text overlays within the game engine were added in front of the User Interface image in Figure 3, displaying supply chain parameters.

The design consists of a combination of icons and texts which complement each other. Starting from the upper left: the four icons display information regarding the stage number, inventory, previous order amount, and lead time. The icons on the right display information regarding revenue, lost sales, and the current cost of inventory (holding costs). In the middle of the scoreboard, the main overview of the supply chain is given, with each actor represented by a specific icon and colour, with additional boxes that display the order, transit, and inventory amounts. The arrows indicate the direction from or to actors of the order and transit amounts. The player plays the role as an inventory manager for a supermarket, represented by the red rectangle and arrow. Underneath, a 2D plot displays the order amounts that are present in the green boxes via a bar chart. The black line served as a trend line, displaying the flow of orders within the chain.

#### 3.1.2. Game Logic

The manufacturer and the distributor follow a set of rules that is commonly used in supply chain management. These two actors make ordering decisions based on a 95% service level, a reorder point, and the economic ordering quantity principle. To start with the 95% service level, these two actors had to ensure 95% availability of always keeping enough products in stock to serve their customer. A safety stock, as indicated in (1), is required to hit 95% availability, and the next step is to determine at what inventory level the actors should place an order to ensure their 95% availability.

(1) $$SS=z\;\bar{D}\;L$$

where SS is safety Stock, z is the service level factor, $\bar{D}\;is\;the\;average\;demand$, and L is the lead time A reorder point is calculated in (2) for that goal where safety stock is the number of products in stock calculated in the previous formula.

(2) $$ROP=SS+\bar{D}\;L$$

*ROP* refers to the reorder point, *SS* is safety stock, $\bar{D}$ is the average demand and *L* is the lead time. The final step (3) is to determine how many products to order when the inventory levels fall below the reorder point. The Economic Order Quantity (*EOQ*) relates to the ordering and holding cost (both = 1) and differs from the values from the player.

(3) $$EOQ=\sqrt{\frac{2\bar{D}K}{h}}$$

where *EOQ* = Economic Order Quantity, $\bar{D}$ = average demand, *K* = order cost, and *h* = holding cost. Knowing the theoretical underpinning of the game’s objective and NPC’s behaviour, the next steps consist of implementing the game logic in C#, creating 3D models and assets, and integrating all game elements in the game engine of Unity (V2019.4.31.f1) with the VRC Software Development Kit (SDK V3.4.2). The VRC SDK is a free-to-use Unity-compatible SDK with built-in support for modern VR headsets, multi-platform compatibility, locomotion, and comfort features.

### 3.2. EEG Sensor and Experiment Processes

In total, 35 participants signed up for the experiment, promoted through social media, posters, and direct invitation using convenience sampling. The aim was a preparation time of 5 to 10 min per participant, with a VR experience lasting 15 to 20 min, during which the participants from both groups experienced (while in VR) a short tutorial, a 3 min virtual SCM lecture, and the supply chain role-playing game, playing as an inventory manager for a grocery store. To eliminate movement artifacts, participants played the game whilst sitting down, and used the joysticks for navigation so their head remained still.

#### 3.2.1. VR Equipment and EEG Sensor

**Figure 4:**
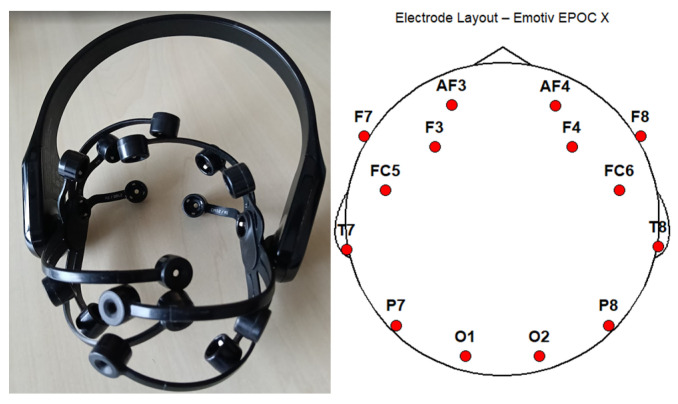
(**Left**) EEG headset by Emotiv (based in San Francisco, USA) and (**Right**) electrode layout.

A Meta Quest 3 (build V60) was used in combination with an Emotiv Epoch X EEG headset (Figure 4). A desktop PC with an Oculus Link cable—running SteamVR (V2.3.1), Oculus Link (V3.1.13), and VRChat (V2023.4.2)—was the primary computer for rendering the experiment. The application EmotivPRO (V3.0.4) recorded the EEG data on a separate laptop via the included dongle during the experiment.

Specifically, the EEG procedure gathered neurocognitive responses during specific time intervals: specifically at the start of a 3 min virtual introduction lecture (time event 3) and 5 min of playing after round 1 (time event 4; tutorial). Time event 1 and 2 were collected as calibration and baseline measurements (eyes open and eyes closed). This data consisted of 14 frequency bands related to the number of electrodes placed on the participant’s head. EEG is a non-invasive method that measures the electrical activity from a group of neurons through an EEG electrode that is placed outside the scalp. When a neuron becomes active, electrically charged ions travel through the dendroid and axions of the neuron. This in- and out-flow of ions have a spatial organisation, generating an electrical field that can be detected by the EEG electrode. The electrical activity that is generated by the membrane of a single neuron is too weak to detect for an EEG electrode.

However, many neurons become active simultaneously, generating an electrical field collectively strong enough to be detected by the electrode. This signal is amplified and stored, creating voltage fluctuations (voltage deflections) per electrode in milliseconds. Raw EEG data consist of voltage reflections of instantaneous activity of clusters of neurons. Thus, this section discusses the workings of EEG in the format of a general overview of how the data are generated [22]. For this, the electrodes were named AF3, F7, F3, FC5, T7, P7, O1, 02, P8, T8, FC6, F4, F8, and AF4. The structure of the electrodes follows the standardised 10–20 EEG naming convention system. Each electrode can contain one or two letters and indicates the topographical region. F stands for frontal, C for central, T for temporal, P for parietal, and O for occipital. The numbers indicate the right (even numbers) or left (odd numbers) hemisphere of the brain and increases the further away they are from the centre. Odd numbers indicate the left hemisphere, while even numbers indicate the right hemisphere. The preprocessed power-band values collected from EmotivPRO was analysed in MATLAB (V2023b) and R (V4.3.2) to generate topographic heatmaps for comparison between the two groups. These topographic heatmaps are validated through Neurosynth, which offers automatically synthesised active patterns from neuroimaging studies to identify brain–cognitions relations. EmotivPRO offers automatic preprocessing and power-band calculation. Specifically, (1) Emotiv applies automatic trial rejections with datapoints that have high artefacts (e.g., movement and interference), (2) EEG signals are filtered into frequency bands (theta, alpha, beta-low, beta-high, and gamma) and (3) Fast-Fourier Transformation is applied to calculate the absolute power-band values per electrode. The preprocessed data (in .edf format) are imported in MATLAB using the MATLAB EEGLAB package. After processing, the results are formatted in Excel and imported in RStudio (V2023.12.1+402), where the topographic heatmaps are created using the eegUtils (V0.7.0) package.

In addition to measuring the raw frequency data, Emotiv, through EmotivPRO, also provides performance metrics. Namely Excitement (Arousal), Interest (Valence), Stress (Frustration), Engagement/Boredom, Attention (Focus), and Meditation (Relaxation). Each metric provides insights into how the participant is performing in that area. For example, the attention metric evaluates the attention span of the participant on a specific task with a normalised score between 0 and 1, indicating whether their focus on that task is good (high value) or poor at that moment. Table 1 provides a brief overview of the meaning behind each performance metrics. The attention (or focus) metric played a central role as the primary metric in this study, aligning with the objectives and research questions that guided this research investigation. The PM values will be analysed in R, with normality (variance, normal distribution) checks to determine the optimal statistical test to compare the distribution differences between the two groups. It should be noted that Emotiv does not offer transparency on their exact PM calculations, leaving it open for interpretation, but they do provide a synopsis into their power-band calculations. Reverse engineering is therefore possible; see [23] for the reference script.

#### 3.2.2. Survey Process

In total, two surveys were created—a pre-survey (or sign-up sheet) and a post-survey after completion of the VR experiment. Starting with the selection of the participants, the main target group were students in higher education, above or at the age of 18. The main distinction that was made between the participants was the (non) identification or diagnosis of ADHD. These two diagnoses were used to increase the potential numbers of ADHD participants.

The pre-survey was distributed via Qualtrics and is detailed in Table 2. The twelve questions were for participants that identify or were diagnosed with ADHD. Participants without ADHD received eleven or ten questions instead, depending on the responses on the ADHD diagnosis and their previous experience using VR due to follow-up questions. A five-point Likert scale was used, regarding the participant’s familiarity with the term VR. The results are analysed in R with a permutation and Mann–Whitney U (Wilcoxon) non-parametric test, following the normality checks, to compare the statistical differences (median and shift distribution) between the two groups.

To ensure anonymity and the privacy of the participants, a unique identifier (ID) was generated in Qualtrics per participants that consisted of the first two letters of their first name and three random generated numbers—this is a requirement for the Research Ethics Committee who approved the study. A mixed approach was taken with the questions asked during the experiment, with some asked while the participants were in VR and a subset after completing the VR experiment. According to related works by Safikhani et al. and Alexandrovsky et al. [24,25], the in-VR questionnaire provides comparable results as outside-VR questionnaires, but participants experience a higher enjoyment. For the experiment survey questions in Table 3, the questions consisted of a combination of the Igroup Presence questionnaire (IQP), the Technology Acceptance model, and questions created by the researcher. The questions regarding the Presence and Technology Acceptance model (TAM), were inspired by Marangunić et al. [26] and were chosen to categorise the experiences of the participants into multiple facets that relate to the usefulness of the technology. The Presence Questionnaire has been a popular questionnaire in assessing the presence of participants in a virtual environment for many years [27]. It consists of fourteen items that are divided into four sub-categories. Namely, General Presence (GP), Spatial Presence (SP), Involvement (INV), and Realism (REAL) [27]. The Igroup Presence Questionnaire has been the most appropriate questionnaire to use for measuring presence in VR.

Based on the works by Schwind et al. (2019) [27], the IQP scored the highest on inter-correlations among the items of the questionnaire compared to the Slater–Usoh–Steed (SUS) and the Witmer and Singer Presence (WS) questionnaire. The effectiveness of immersion and presence in the virtual environment were evaluated using the IQP questionnaire to evaluate if the environments prove successful in being an engaging and effective learning environment. The TAM and IQP provide an understanding of the ease of use and perceptions, along with the presence of immersion and engagement, of the participants.

The TAM has been a widely used framework to assess the potential acceptance or rejection of a given technology, originating from psychological theory of reasoned action and the theory of planned behaviour. The perceived ease-of-use and usefulness of the VR technology was incorporated in the survey questions to provide an overview of the acceptance of VR among the participants.

The questions were a combination of open and selected choice questions. The selected choice questions used a 7-point Likert scale. This scale has been used, as opposed to the five-, six- or ten-point scale, due to its preferred significance and statistically higher relevance [28]. The remaining questions were open questions, providing the participant the opportunity to give a more in-depth, personalised answer.

The attention (or focus) metric played a central role as the primary metric in this study, aligning with the objectives and research questions that guided this research investigation. The other metrics were stored too, to identify additional explanations of user’s actions and their responses in VR. The aggregated data over time will be displayed per group, along with a summary of the characteristic features for each performance metric.

## 4. Results

In this section, we provide a descriptive overview of the participants, followed by the analysis of the experiment survey. A comparison of the performance metrics is given, followed by a visual representation of the topographic heatmap from the EEG data.

### 4.1. Participant Overview

Both groups combined totalled n = 35, of which n = 14 for the experimental group and n = 21 for the control group. The average age of both groups is comparable, indicating that the majority of participants were in their mid-twenties, as indicated in Table 4.

Furthermore, the majority of the participants were male, with the control group having a higher percentage of females compared to the experimental group. The participants were dominantly students or employees at the Wageningen University (Table 5), with a few exceptions of students or employees outside the university. One participant was not active in an education setting due to a recent graduation. The experimental group consisted of participants that had ADHD and included a blend of individuals with an official diagnosis or identification, as summarised in Table 6. A total of 35 participants completed the full experiment, producing complete EEG data suitable for analysis. Data from n = 5 additional participants were excluded before any preprocessing or analysis steps was performed primarily due to poor electrode contact caused from unsuitable head dimensions, inaccessibility to the scalp, or VR headset interference.

### 4.2. Participant Perception of the VR Experience

**Figure 5:**
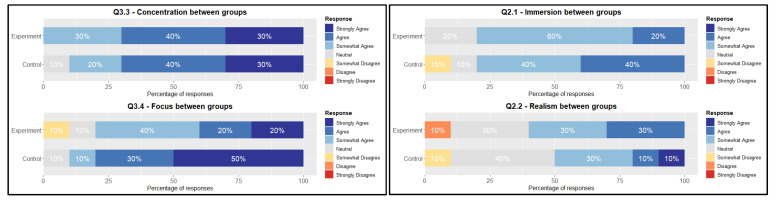
(**Left**) Likert overview Question 3.3 and 3.4, and (**Right**) Spatial Presence between both groups.

The majority of the participants from both groups were very familiar with the concept of VR, but most had not tried the technology prior to the experiment. However, the response from both groups favours the use of VR for improved concentration purposes, with the majority responding with “agree” on Question 3.3 (Figure 5 (Left)). The average response exhibited a positive sentiment in 95% of cases, indicating a strong inclination towards agreeing. There is no significant difference between the experimental and control group, albeit the control group responding slightly less positive, with 90% positive and 10% neutral. The results from Question 3.4 display a similar pattern for the control group, with more responses towards “strongly agree”, shown by the 90% inclination towards agreeing.

The experimental group was also positive on the use of VR on their ability to focus, with the majority responding with “somewhat agree”. There is a slightly more negative response, but the offset is toward the agreeing side with 80%. No significant difference between the median or the distribution shift has been found. The spatial presence (Figure 5 (Right)) comprises two questions regarding immersion and realism. Starting with immersion, both groups “somewhat agree” with their perceived immersion level while playing the game, indicated by the 80% inclination towards agreeing.

There is tendency in the control group towards the “agree” option, albeit with a minor margin. Realism tends to have mixed responses in both groups. The majority aims towards a positive sentiment, by “somewhat agreeing”. However, there is a number of participants responding with “neither agree nor disagree”, indicated by the 30 and 40% neutral responses between the experimental and control group, respectively. A portion of participants disagreed with the realism of the experience. No statistical difference in a distribution shift between groups or a difference in median was observed (Table 7). In summary, for both groups, the spatial presence was perceived as quite high, with the majority of responses from questions 2.1 and 2.2 resulting in a positive sentiment regarding immersion and realism with 80% and 55% on average, respectively, between the two groups.

**Figure 6:**
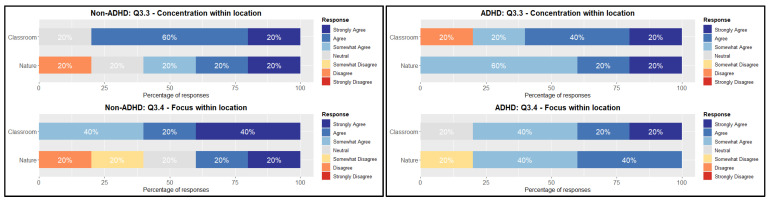
Likert overview Question 3.3 and 3.4 within groups.

In Figure 6, the perceived attention for the experimental group is mostly perceived as positive in both locations, with 90% of the responses focusing on positive sentiments (Question 3.3: Concentration ADHD). The classroom environment, although having a single negative response, is dominantly responding with “agree”. This indicates that both environments are perceived positively towards contributing to the attention span of the participants.

The control group follows a similar path, although there are more neutral responses in both environments. The classroom environment scores the highest on the positive sentiment compared to the nature environment. The findings indicate that there are minimal distinctions between the two groups and settings, with the classroom setting slightly outperforming the natural environment in terms of perceived attention (Table 8).

Reflecting on the perceived aptitude for focus inquired about in Question 3.4, it is clear that there are no significant variances between the two locations for the experimental participants (Table 9). The classroom environment has an edge over the nature environment with more responses being “strongly agree”. The control group on the other hand shows a shift in the responses, with a 40% (*n* = 2) distribution among the positive and negative sentiment responses, compared to the 100% positive sentiment in the classroom environment. However, in both questions, there is not a statistical difference in the distribution shift within the groups or a difference in median.

### 4.3. EEG Data Analysis

**Figure 7:**
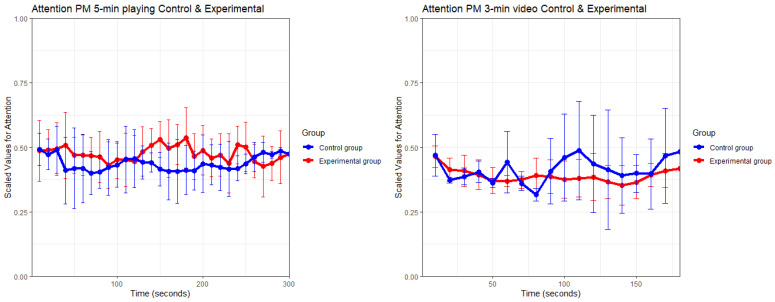
The aggregated data of time event 3 and 4 between both groups of the attention PM.

This section documents the results from the performance metrics (PMs) obtained after analysing the data from the EmotivPRO software (V3.0.4). Figure 7 illustrates the aggregated data of the scaled attention performance metric from two-time events: 5 min of playing and watching a 3 min virtual lecture. This analysis includes all participants with high-quality EEG data (experimental = 9, control = 6), defined as continuous recordings with consistent contact quality (CQ) and EEG Quality (EQ) values generated by the Emotiv system above 70%. An interpolated method has been applied to suppress the influence of missing values, but no major differences in the patterns were found, hence the use of the original plot data. Assumptions of normality have not been met in both time events, resulting in using the Mann–Whitney U non-parametric test to compare the PM differences between the two groups.

Starting with Figure 7 (Left), of time event 4, over the duration of 300 s, the experimental group consistently scores a higher attention PM score compared to the control group. Only during the last 30 s of the time event dips the aggregated line of the experimental group under its neighbour. The error bar indicates there are some fluctuations among both groups, especially the first 140 s. It indicates that some participants of the control group score higher on the attention PM during this part of their experience. Nevertheless, the primary pattern shows that the experimental group demonstrates a higher performance score in attention when compared to the control group. Within the attention score, an important factor to consider is the impact caused by excessive task-switching. It illustrates poor focus and distraction. The gathered medium to high scores on this metric indicates that the participants were mostly indifferent to this negative influence on the score.

Figure 7 (Right) illustrates an opposite pattern. Most of the duration depicts a very similar score among both groups. Halfway through the experience, the attention score from the control group spikes and overtakes the experimental scores. The error bar indicates that there are quite some fluctuations within the result, especially compared to the relatively stable error bars from the experimental group. Although the pattern follows a very identical path for the first half of the time event, the second part shows an increase in attention (spikes) among the control group. Reasons for the increase are speculative, but one possible explanation is the shift in the video’s content midway through.

Specifically, at around 75 s, the video provides an explanation of the practical aspects of the game, such as supply chain parameters and tips on how to play the game. Table 10 provides an overview of the descriptive elements from the six performance metrics that were recorded during the two time events (5 min of playing and watching a virtual lecture).

Table 10 confirms the pattern seen in Figure 7 for the playing time event. The experimental group has a higher mean value for five performance metrics compared to the control group, with one exception: the engagement metric. The engagement metric illustrates artifacts (constant, unusually high values) in the signal for the control group which was likely caused by a defective electrode or bad contact, rendering it unusable for comparisons. These EEG results were excluded in the final analysis, resulting in the smaller sample size. Nevertheless, the engagement value displayed consistent higher values compared to other PMs, likely caused by the immersive nature of VR. Similar findings were found in a paper by Paranthaman et al. (2021), although participants’ self-reported measures on engagement did not always align with the PM value [29]. The experimental group does have a higher standard deviation across the board, indicating that the spread differs quite heavily. For example, the excitement metric has a relatively large deviation, with even higher deviations for the stress, relaxation, and interest metric.

The engagement score indicates a high alertness and conscious direction towards the task at hand, whereas the stress score signifies a level of comfort that is possibly a result of an unsuccessful task completion. This was particularly true when the participant felt that they were making mistakes in the ordering process of the supply chain game. In general, the experimental group was very engaged during the 5 min of playing. The Mann–Whitney test indicates that 5 PMs, except for the (non-interpolated) interest PM, show a significant shift in distribution between the two groups.

The interest, attention and stress level were relatively high during this time, which explains the lower relaxation level too. Although the participants had the option to manage the experiment according to their own time preferences, the inherent nature of the game likely encouraged the participants to remain consistently active and engaged, rather than taking a break even for a brief moment, as indicated by the increased engagement score. The control group follows a similar trend, albeit with lower values and a lower standard deviation across the metrics. The high interest values for both groups indicate a high level of attraction towards the current stimuli, illustrating that the participants had a strong affinity to the learning task that was presented in front of them.

The values in Table 11 for the virtual lecture time event indicate that the mean values from the experimental group for all metrics are lower compared to the control group, indicating less stimulus during the watching of the video compared to the playing element for the experimental group. The control group on the other hands scores higher compared to the experimental group, but still shows lower scores overall compared to the result from time event 4. Nevertheless, four PMs, except for attention and interest, show a significant shift in distribution. In summary, the experimental group exhibited a higher level of engagement and interest, suggesting that the video task successfully captured their attention. However, it is worth noting that the participants did not display significant excitement or attention, indicating a lack of physiological arousal during the video viewing.

This finding is further supported by the low attention score, which might indicate a lack of sustained focus on the video. Although the video only required the participant’s passive engagement, the low relaxation and stress score indicate that the video was not particularly relaxing nor stress-reducing. The low relaxation score falls in line with its ability to detect the participants capacity to unwind and recover, which was not evident during the course of the experiment. The same patterns can be explained to the control group. A difference between the scores from the control group are the slightly higher scores overall, in particular, the higher excitement and interest scores. It shows more engagement and stimuli from these participants during the watching of the video, as opposed to the experimental group. The video also illustrates a classic approach of an existing lecture format, with the presentation of information through PowerPoint slides which differs from the interactive 5-min playing part of the experiment.

**Figure 8:**
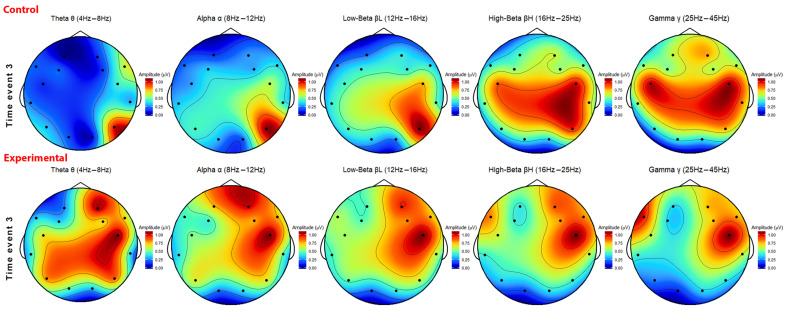
Topographic brain activity heatmap of time event 3: tutorial video between control group (upper, n = 3) and experimental group (lower, n = 9), in five frequency bands (from left to right: θ, α, βL, βH, and γ). The average power band values of the 3 min time event are normalised (blue to red: 0–1).

**Figure 9:**
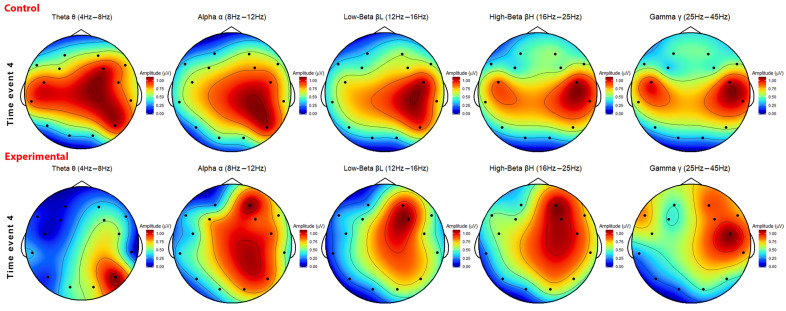
Topographic brain activity heatmap of time event 4: 5-min playing between control group (upper, n = 6) and experimental group (lower, n = 9), in five frequency bands (from left to right: θ, α, βL, βH, and γ). The average power band values of the 5 min time event are normalised (blue to red: 0–1).

Figure 8 and Figure 9 depict the aggregated power band values from both groups for time event 3 and 4. To start with time event 3 in Figure 8, the control group shows different brain activity across five frequency bands. On average, the prefrontal and frontal areas of the control group have less activity compared to the experimental group. A common trait is the centred activity between the right central and right temporal cortex between both groups. The occipital area and the left parietal cortex from both groups show the least activity, with a slight increase in activity for the experimental group on frequency band θ, α, and βL.

The high-beta wave frequencies (16–25 Hz), often associated with high levels of concentrations, and the gamma frequencies (25–45 Hz), often associated with perception and problem-solving, show the most spread-out dominant activity. On the other hand, the theta, alpha, and low-beta frequencies are predominantly active in the right parietal cortex.

The experimental group shows the most activity in the right central, prefrontal, right frontal, and right temporal cortex. All five frequencies are distributed among these areas, with the theta frequencies (4–8 Hz), often associated with relaxation during activities that involve visualisation and creativity, being spread out the most. To illustrate the difference in concentration, the low-beta and high-beta frequencies of both groups were cross-checked with Neurosynth. Neurosynth is a platform designed for automatic, synthesised neuro(imaging) findings, employing a quantitative approach. The Cartesian coordinate system, a common system to display the 10–20 naming convention, has been converted to the standardised MNI152 XYZ coordinate system with the use of the LORETA-KEY software (V2003-June). The MNI152 format is a 3D standardised template for neuroimaging research and represents an anatomical average representation of the human brain. The coordinates are adapted to accommodate the external placement of electrodes on the scalp suitable with Neurosynths’ system.

In the control group, the right parietal cortex has the most activity of low-beta frequencies in. Electrode P8 indicated the most dominant activity for all frequencies; with P8 located at the right posterior parietal region, an area that is responsible for visual and spatial awareness.

The motion and visual centre being active indicates that a big portion of the brain activity from the participants is devoted to the processing and perception of visual information. This makes sense, knowing that VR and a lecture given in video format requires visual processing of the presented information. Throughout both time events and across both groups, this area of the brain shows a constant activity across all frequencies, indicating that VR usage results in a generally high activity in the right parietal cortex.

Electrode FC6, located in the prefrontal area near the right frontal lobe, and electrode FC5, located in the prefrontal area near the left frontal lobe, are generally dominant for the beta frequencies as well. Starting with electrode FC6, located in [12,34,52], associations commonly found for this area of the brain are the premotor function, parietal lobe, and the ventral premotor. Premotor functions of the brain are responsible for the coordination, organising, and planning of movements or actions. In addition to its involvement in sensory processing, perception, and integration, the parietal lobe is responsible for the coordination and management of taste, hearing, vision, touch, and smell. The ventral premotor plays a crucial role in combining phonological processing, such as language processing. The activity around this region for both groups suggest activity in motor functions and language processing, a logical outcome given the inherent nature of watching a lecture video in VR at this particular moment.

Activity at coordinates [−52,12,34] of electrode FC5 is highly associated with the phonological process, frontal lobe, and ‘demand’. The phonological process is responsible for the understanding of language through incoming sounds. The frontal region of the brain is involved in reasoning, planning, decision-making, and some motor functions. Demands refer to the brain’s cognitive capacity to generate necessary prerequisites for the processing of auditory stimuli to facilitate language comprehension. The high-beta frequencies across electrode FC6 and FC5 suggest that the majority of the participant’s brain function was dedicated to language processing, with additional involvement in cognitive functions such as reasoning, planning, and learning in response to auditory stimuli.

The experimental group display both similarities and differences to the control group. Notably, both groups exhibit a high level of activity in electrode FC6 for both low and high-beta frequencies. The findings suggests that the cognitive mechanism responsible for language processing was particularly active during time event 3. However, a difference was observed in the reduced stimuli within the visual centre at the back of the brain. While activity was happening, it was less prominent compared to the control group. Another difference is the high activity across multiple parts of the brain among the theta frequencies. Theta frequencies are associated with creativity, visual stimuli, and problem-solving. In comparison to the control group, there is an elevation in activity observed in memory-related electrode FC6 situated the right frontal lobe. Observations of electrode AF4, situated in the right prefrontal cortex, display increased activity as opposed to the control group. Electrode AF4 at location [30,50,36] exhibits a strong connection with the cortex dlpfc (or dorsolateral prefrontal cortex (DLPFC). The dlpfc plays an important role in working memory, decision-making, planning, and abstract reasoning [30]. Notably, the experimental group shows more activity in this specific area, indicating elevated levels cognitive functions related to memory and decision-making. In contrast, the control group did not display increased activity in this region. This observation may suggest that the experimental group demonstrated higher engagements during time event 3 due to increased cognitive functions linked to memory and decision-making functions.

Electrode P8 at the right parietal, as mentioned earlier, and electrode P7 (coordinates [−52,−73,12]) display increased activity. These electrodes are positioned over the occipital/parietal part of the brain, which plays an important role in visual processing. Electrode P7 is predominantly linked to the extrastriate cortex, which is responsible for higher-order processing of visual signals. This indicates a complex system of analysing visually presented objects [31]. The increased activity observed in the extrastriate cortex might explain the higher order processing of the information presented in this time event. Although electrode P7 is associated with processing of visually presented objects, in this case, the objects are virtual, computer-generated images through the headset. Nevertheless, both groups show increased activity in these areas, albeit less pronounced in the control group.

A final observation is the observed heightened activity in electrode F7, located in the left temporal / left frontal lobe of the brain. The heightened activity is most noticeable in the high-beta and gamma waves, suggesting that these frequencies indicate higher levels of active concentration and advanced cognitive processes. Electrode F7 at coordinates [−52,32,2] is primarily linked with semantic and language processing. While the control group displays some activity in this area, it is less pronounced compared to the experimental group.

In general, time event 3 displays similarities, particularly in the areas of motion, vision, and language processing areas of the brain. Differences between the groups are evident in the increased activity near electrodes AF4 and F7 in the (pre)frontal cortex in the low- and high-beta frequencies. This might suggest increased levels of memory-related and language processing functions as opposed to the decreased activity observed in the control group. The heatmap displayed in Figure 9 illustrates similarities and differences in brain activity observed between both groups during time event 4. The control group displays the highest activity in the right central and right temporal areas of the brain. Moreover, moderate activity is observed in the left central and right parietal brain regions. The beta and gamma frequencies in the control group are predominantly active in the right central part of the brain, although there are outliers near the left central part of the brain. Similarly, the lower frequencies follow a similar trend, but with the inclusion of activity in the right parietal area, particularly in the theta frequency.

The experimental group displays a higher activity in the right hemisphere, with peaks near the right frontal cortex and the right parietal lobe. The beta and gamma frequencies demonstrate most activity concentrated near the frontal cortex, while the lower frequencies also display activity in the right parietal region of the brain. In contrast, the theta and gamma frequencies deviate from the overall pattern in the experimental group with clustered activity observed in the right parietal and left frontal, respectively. Both groups share a common characterising of recurring activity in the right parietal lobe through the visual centre electrode P8. This suggests that that the motion and visual processing area of the brain remain very active during a VR session. However, a difference between the two groups lies in the increased activity observed in the left hemisphere of the control group, whereas the experimental group displays the most dominant activity in the right hemisphere of the brain.

To investigate the attention and concentration differences further, the Neurosynth website was utilised to compare the low- and high-beta frequencies between the two groups. Starting with the control group, increased activity can be observed in the area around electrode P8, which is responsible for the visual and motion function of the brain, particularly in the right parietal lobe. This observation was not only observed for time event 4 but is also prominently present during time event 3 in both groups. The reason might be attributed to the visual stimuli that happen during a VR experience where participants control their viewpoint in the virtual space by physically moving their head around, all while simultaneously processing the presented information in front of them.

Increased activity can be observed around electrode FC6, which is responsible for the premotor functions of the brain, particularly during time event 4, when participants were required to interact with virtual items, press buttons, and move their head towards specific areas of the environment to advance further in the game. Both groups displayed increased activity, suggesting that motor functions were actively engaged during the experience. The right temporal lobe area displays increased activity in the beta frequencies for the control group. Electrode T8, positioned at [64,−18,8], is the designated electrode in this region and is primarily associated with the auditory cortex and speech functions of the brain. The experimental group also display increased activity in this area, in particular for the high-beta frequencies, suggesting that sound processing played a role during time event 4. Additionally, a cluster of activity can also be observed around electrode FC5, which is responsible for the phonological process and is located around the frontal lobe. The observed activity in this area might suggest increased involvement of the language processing ability, as well as the reasoning, planning, and learning ability of the brain.

The experimental group displays similarities in brain activity around the parietal part of the brain, specifically around electrode P8 and O2, which are responsible for visual processing function. Differences can be observed in the increased activity in the frontal and prefrontal regions of the brain, particularly around electrodes AF4 and F4. Electrode AF4 is responsible for the working-memory function of the cortex dlpfc mentioned previously. Electrode F4 in the right frontal lobe at coordinates [36,34,50] is associated with the Locus coeruleus, aversive stimuli, and the cortex anterior (or anterior cingulate cortex). The Locus coeruleus, a densely packed cluster of cells within the noradrenergic system, is associated with functions such as learning, memory, attention, and other higher-order processes such as decision-making and uncertainty [32]. The region is particularly active during interruption of behaviour, such as a shift in environmental demands due to novel stimuli or changes in consequences during specific stimuli, seen in the VR supply chain game (bullwhip effect, lost sales, etc). The activity in this area of the brain may explain part of the learning and adapting process observed in the (experimental) participants while engaging in the supply chain game and making decisions based on evolving parameters of the game. Additionally, electrode F4 is linked to aversive stimuli, negative responses that are unpleasant, and the cortex anterior, which is involved in attention allocation, reward anticipation, decision-making, and impulse control [33].

In addition to the specific areas mentioned previously, the (right) frontal cortex also exhibits a moderate level of activity. This level of activity is especially notable when compared to the comparatively lower activity in the control group within the same region. The frontal cortex is responsible for various cognitive functions, including reasoning, planning, problem solving, learning, and more. In general, the experimental group displays increased activity in brain regions associated with attention, memory, and decision-making. These findings align with the learning objective of the supply chain game, which revolves around learning through the process of decision-making, strategic planning, and effectively managing a supply chain. Although the control group displays similar activity in the frontal cortex, it is less pronounced compared to the experimental group. Instead, the control group primarily displays clustered activity in areas responsible for the visual and motor functions, followed by sound and language processing regions of the brain. Nevertheless, clear differences between the two time events and the activity patterns among both groups are displayed in the heatmaps.

### 4.4. Discussion

The consensus of deploying VR for learning purposes is that the participants had a positive experience and noted its potential. The qualitative results indicate that the participants foresee a future of using VR for learning purposes, particularly with the experimental group showing a stronger drive towards VR adoption. The VR experience offered the participants with an enjoyable and interactive learning experience, simultaneously aiding their understanding of a rather intangible concept. Examination of the EEG data revealed that both groups displayed high levels of engagement, interest, and attention, indicating their active involvement and sufficient focus towards the subject. Notable, the experimental group displayed more pronounced effects in the EEG data, signifying increased engagement of memory, attention, and decision-making regions of the brain. This suggests that the VR experience effectively sustained engagement and mental activity. The levels of concentration and focus further support this observation. Participants frequently attributed the (mostly) absence of distractions and the interactive and gamification elements within the VR experience as key factors that contributed to their elevated engagement and activity levels. However, it is important to acknowledge that the high stress and low relaxation levels suggests that the experience may have been mentally taxing for some participants.

In addition, data quality and signal integrity are important considerations when combining VR with EEG. Electronical noise from the VR headset can contaminate EEG recordings, as well as improper electrode contact and movement artefacts. The flexibility of the Emotiv Epoc X helps with rapid setup; however, its use in combination with a Meta Quest 3 HMD resulted in frequent data artefacts and loss of electrode contact. While EmotivPRO provides automatic trial and noise rejections, as well as data quality metrics in their software, a large portion of the collected EEG data proved to be unusable due to signal noise, loss of contact, and movement artefacts during the trial, hence the lower number of EEG analysis. Future experiments should consider the use of a fixed EEG cap to improve electrode contact in combination with a VR headset. Nevertheless, lower sample sizes are common in neuropsychological research and can still offer valuable insights, if the paradigm is developed properly, considering the repetition of trials [34].

The Emotiv Epoc is a widely used consumer-grade EEG headset, but the results should be interpreted with caution, as it is classed as a non-medical sensor device. Yet, there are several studies showing that the Emotiv EEG recordings produce results that are comparable to professional BCIs, indicating that the headset can provide valuable insights [35,36]. However, the PM values calculations in the EmotivPRO software (V3.0.4) and limits the interoperability and reproducibility of the values, as noted by related studies utilising an Emotiv BCI [35]. This makes the interpretation of the results difficult. Emotiv does provide a synopsis for their power-band calculations with the raw EEG data (as used for the topographic heatmaps) but has not published the underlying assumptions and preprocessing steps for their PM outcomes. A reverse-engineered script for generating the power-band values with parameters is provided by Emotiv.

While the VR environment offers more control compared to real-world observations, careful considerations into designing VR experience and educational content is necessary to create effective experiences for the students. The lack of clear guidelines on how to convert educational topics into VR learning experiences and how to develop combined VR and EEG studies limits reproducibility and applicability to other academic domains. Despite not being the focus of this paper, we recommend applying user-centric designs that mitigate health concerns (accessibility features such as teleportation/locomotion principles), multi-platform compatibility (e.g., access to the content via phones or laptops), and alignment with learning objectives (edutainment) to maximise user engagement and useability with (intangible) educational topics. A recently published guideline to combine more sophisticated EEG tools with VR might be a first step in this endeavour [20]. Still, additional research is required to explore potential solutions to mitigate these concerns.

#### 4.4.1. What Was the Effect of the Environment on Effective VR Learning?

A high level of learning effectiveness, concentration, and focus was evident in both environments. Although no significant difference was found in the outcomes of the qualitative results between both environments across both groups, the nature environment did show a slight advantage in terms of higher perceived concentration and focus levels when compared to the classroom setting. It is worth mentioning that some participants expressed experiencing pressure to perform in the classroom, which could explain the slight lower perceived concentration experience for students with ADHD. Nevertheless, both were found to be effective for facilitating a learning environment and did not display any significant variances between the groups.

#### 4.4.2. Did VR Provide Attention-Span or Learning Improvements for the ADHD Students?

Improved attention, concentration, and focus levels were observed in both groups during the experiment. The EEG data analysis revealed that the experimental group displayed high attention, engagement, and interest scores while participating in the 5 min of playing. However, it is worth noting that the performance metrics for this group consistently displayed lower scores during the virtual tutorial video in time event 3. The topographic heatmaps showed increased voltage potentials in the right frontal and prefrontal lobes of the brain. The validation of dominant activity near electrodes in the heatmap suggested elevated engagement in areas of the frontal cortex responsible for attention, memory, and decision-making. Throughout the experiment, brain activity of the participants exhibits continuous activity in multiple regions, including the frontal and prefrontal areas. Additionally, there is also significant activity in the occipital and temporal areas, which area associated with visual processing, motor control, and language comprehension. This suggests that the brain exhibits influential activity in regions beyond those that are typically linked to attention-related functions.

## 5. Conclusions and Future Work

VR offers opportunities that have the potential to revolutionise the future of education, but it does come with challenges and obstacles that hinders mainstream adoption. This study focuses on an educational VR case study that explores the impact of attention and learning among students with ADHD. The participants experienced improved attention, concentration, and focus levels as evidence by the qualitative findings. They also demonstrated increased engagement and motivation to learn, attributed to the interactive gamification and immersive elements in the VR environment. These subjective outcomes were systematically validated through an EEG-based multimodal sensing pipeline, enabling sensor-driven data acquisition, processing, and interpretation of neurophysiological signals within an interactive, education-driven VR system. This study provides valuable insight into the use of VR for neurodiverse students, supported by quantitative EEG data collected through a BCI. Thus, this work contributes to the broader field of sensor-driven human–robot and HCI by demonstrating how physiological sensing can be embedded within VR-based educational-driven simulations to objectively evaluate user attention, engagement, and cognitive states. The findings highlight how inclusive interactive system design can leverage multimodal sensing to adapt immersive environments to the needs of neurodiverse users in higher educational settings. It highlights how design practices and learning theories can be incorporated into the development of VR-driven learning environments, providing practical insights on design elements that can address barriers to the adoption of educational VR applications, particularly addressing health-related issues. There is no one-size-fits-all solution of VR development that is meant for education, leading to practical obstacles such as development tools, practices, and financial investments, that hinders adoption. Future studies can continue investigating how VR design (e.g., use of teleportation, optimisation, and realism) affects specific subsets of students that could benefit from a VR learning approach compared to the convention learning system. The proposed approach illustrates how sensor-informed design decisions can mitigate usability, health-related, and adoption barriers across immersive applications, whereby the findings from this research highlight the effectiveness of alternative learning methods for neurodiverse students, extending the application of EEG-driven evaluation frameworks to inclusive, sensor-enabled immersive systems beyond traditional learning contexts.

## Figures and Tables

**Table 1:** Performance metric overview.

Metric	Description
Attention	The ability of the participant to focus on one specific task. The depth of their attention is measured, combined with the frequency of their focus switching between other stimuli. A high frequency illustrates poor focus and distraction, which influences the score negatively.
Engagement	Illustrates the participant’s attention and alertness towards a certain task-related stimuli and measures the level of immersion, attention, and concentration. A higher score indicates high levels of these measures.
Excitement	A high score indicates an increase in the physiological arousal of the participant. Measures the physiological arousal of the participant, which is characterised by a range of physical changes through the body, such as pupil dilation, increased blood flow, and an increase in muscle tightening.
Stress	Measures the participant’s comfort towards a given task or challenge. A high score indicates that the participant is overwhelmed and fears negative consequences of failing the current challenge.
Interest	The degree of attraction towards a certain stimulus. A high score illustrates a strong attraction towards a specific stimulus.

**Table 2:** Pre-survey questions.

#	Description	Type
1	What is your first and last name?	Open
2	What is your email address?	Open
3	What is your age?	Open
4	What is your gender?	Selected Choice
5	What is your nationality?	Open
6	Are you a student/employee of a university?	Selected Choice
7	Are you diagnosed with or identify as having AD(H)D or ADD?	Selected Choice
8	Do you take medication or use therapy for AD(H)D or ADD?	Selected Choice
9	How familiar are you with the term “virtual reality”?	5-point Likert
10	Have you ever tried or experienced virtual reality?	Selected choice
11	Did you feel (motion) sickness while or after experiencing virtual reality?	Selected choice
12	Is there anything else you would like to add?	Open

**Table 3:** Experiment survey questions.

#	Question		Theme	Type
1a	How familiar are you with gaming?	-	Player performance	Likert
1b	Taking a look at the environment, can you describe to me what it reminds you of?	-	Player performance	Open
2.1	How immersed did you feel in the virtual environment?	IPQ	Spatial presence	Likert
2.2	How realistic do you find the environment?	IPQ	Realism	Likert
3.1	How involved did you feel while playing the virtual reality role play?	IPQ	Involvement	Likert
3.2	Did you feel that you were losing awareness of your presence in the physical surroundings?	IPQ	Involvement	Open
3.3	How well could you concentrate on the assigned tasks of the role play?	IPQ	Involvement	Likert
3.4	How easy was it to stay focused?	IPQ	Involvement	Likert
4.1	How well did VR assist you in giving a more engaging experience (compared to the real world)?	IPQ	Immersion	Likert
5.1	How emotionally engaged (e.g., curiosity or feeling happy) were you while playing the virtual role play?	IPQ	Engagement	Likert
5.2	Did these emotions impact your ability to learn effectively?	IPQ	Engagement	Open
6.1	How easy did you find it to learn the controls and interact with the environment/?	TAM	Ease of us	Likert
6.2	How straightforward do you think VR is for using it for learning purposes?	TAM	Ease of us	Likert
6.3	Do you find the VR system (equipment and the experience) easy to understand and use for educational activities?	TAM	Ease of us	Open
7.1	How well did VR assist you in understanding the educational purpose (learn about the Bullwhip-effect and managing a supply chain) of this experiment?	TAM	Usefulness	Likert
7.2	Would you see yourself using this technology in the future to assist you for educational learning purposes?	TAM	Usefulness	Likert
7.3	Did VR contribute positively to learning more effectively?	TAM	Usefulness	Open
7.4	Would you argue that this music genre can assist you in learning more effectively?	-	Qualitative	Open
7.5	Did you find the music helpful in learning more effectively?	-	Qualitative	Open
7.6	Were you able to concentrate more effectively?	-	Qualitative	Open

**Table 4:** Summary of Participants.

Characteristic	Experimental Group	Control Group	Total Participants
Total number of participants	14	21	35
Age (mean ± SD and range in years)	26.24 ± 3.22 (22–32)	26.71 ± 4.5 (22–39)	26.43 ± 4.0 (22–39)
Gender distribution	M: 71.4%; F: 21.4%; NB: 7.2%	M: 57.1%; F: 42.9.4%; NB: 0%	M: 62.8%; F: 34.3%; NB: 2.9%

**Table 5:** Educational background.

Characteristic	Experimental Group	Control Group	Total Participants
Wageningen student or employee	71.4%	100%	88.6%
Student or employee elsewhere	21.4%	0%	8.6%
Other	7.2%	-	2.9%

**Table 6:** ADHD identification.

Characteristic	Experimental Group	Control Group	Total Participant
Diagnosed AD(H)D	M: 14.3%; F: 14.3%; NB: 0%	–	–
Diagnosed ADD	M: 14.3%; F: 7.1%; NB: 7.1%	–	–
Identify AD(H)D	M: 28.6%; F: 0%; NB: 0%	–	–
Identify ADD	M: 28.6%; F: 0%; NB: 0%	–	–
VR familiarity (neg/neutral/pos; scale 1–5)	7%/14%/79%	5%/38%/57%	6%/29%/66%
VR experience (neg/neutral/pos; scale 1–5)	57%/21%/21%	67%/24%/10%	63%/23%/14%

**Table 7:** Statistical test qualitative responses.

Question	Result Permutation Test	Question	Result Mann–Whitney U
Q1a (Permutation test)	Z: −0.567, *p*-value: 0.719	Q1a (Wilcoxon rank)	W: 49.5, *p*-value: 1.000
Q2.1 (Permutation test)	Z: 0.271, *p*-value: 1.000	Q2.1 (Wilcoxon rank)	W: 57.0, *p*-value: 0.593
Q2.2 (Permutation test)	Z: 0, *p*-value: 1.000	Q2.2 (Wilcoxon rank)	W: 46.0, *p*-value: 0.783
Q3.1 (Permutation test)	Z: −0.802, *p*-value: 0.539	Q3.1 (Wilcoxon rank)	W: 41.0, *p*-value: 0.501
Q3.3 (Permutation test)	Z: −0.252, *p*-value: 1.000	Q3.3 (Wilcoxon rank)	W: 48.5, *p*-value: 0.936
Q3.4 (Permutation test)	Z: 1.665, *p*-value: 0.140	Q3.4 (Wilcoxon rank)	W: 71.5, *p*-value: 0.099

**Table 8:** Statistical differences locations on median and distribution within the control group.

Question	Result Permutation Test	Question	Result Mann–Whitney U
Q3.3 (Permutation test)	Z: −0.252, *p*-value: 1.000	Q3.3 (Wilcoxon rank)	W: 48.5, *p*-value: 0.936
Q3.4 (Permutation test)	Z: 1.665, *p*-value: 0.140	Q3.4 (Wilcoxon rank)	W: 71.5, *p*-value: 0.099

**Table 9:** Statistical differences locations on median and distribution within the experimental group.

Question	Result Permutation Test	Question	Result Mann–Whitney U
Q3.3 (Permutation test)	Z: −1.009, *p*-value: 0.452	Q3.3 (Wilcoxon rank)	W: 8.5, *p*-value: 0.448
Q3.4 (Permutation test)	Z: −1.445, *p*-value: 0.214	Q3.4 (Wilcoxon rank)	W: 6.5, *p*-value: 0.242

**Table 10:** Performance metrics recorded by EEG.

Performance Metric 5 min Playing	Experimental Group (n = 270; 9 Participants)	Control Group (n = 180; 6 Participants)	Mann–Whitney U Test	Normality (E|C)
Attention (mean ± SD, none)	0.48 ± 0.092 (35 NAs)	0.44 ± 0.086 (3 NAs)	*p*-value: <0.001	*p*-value: 0.002 *p*-value: 0.065
Engagement (mean ± SD, none)	0.70 ± 0.123 (28 NAs)	0.85 ± 0.139 (0 NAs)	*p*-value: <0.001	*p*-value: <0.001 *p*-value: <0.001
Excitement (mean ± SD, none)	0.44 ± 0.256 (0 NAs)	0.38 ± 0.215 (0 NAs)	*p*-value: 0.035	*p*-value: <0.001 *p*-value: <0.001
Stress (mean ± SD, none)	0.54 ± 0.186 (28 NAs)	0.48 ± 0.158 (0 NAs)	*p*-value: 0.006	*p*-value: <0.001 *p*-value: <0.001
Relaxation (mean ± SD, none)	0.49 ± 0.164 (28 NAs)	0.38 ± 0.144 (0 NAs)	*p*-value: <0.001	*p*-value: <0.001 *p*-value: <0.001
Interest (mean ± SD, none)	0.57 ± 0.148 (28 NAs)	0.56 ± 0.142 (0 NAs)	*p*-value: 0.380	*p*-value: <0.001 *p*-value: <0.001

**Table 11:** Descriptive overview time event 3 per PM, non-interpolated.

Performance Metric 3 min Virtual Lecture	Experimental Group (n = 270; 9 Participants)	Control Group (n = 54; 3 Participants)	Mann–Whitney U Test	Normality (E|C)
Attention (mean ± SD, none)	0.39 ± 0.061 (36 NAs)	0.41 ± 0.120 (0 NAs)	*p*-value: 0.785	*p*-value: 0.074 *p*-value: 0.065
Engagement (mean ± SD, none)	0.72 ± 0.109 (18 NAs)	0.88 ± 0.093 (0 NAs)	*p*-value: <0.001	*p*-value: <0.001 *p*-value: <0.001
Excitement (mean ± SD, none)	0.27 ± 0.195 (0 NAs)	0.42 ± 0.246 (0 NAs)	*p*-value: 0.038	*p*-value: <0.001 *p*-value: 0.031
Stress (mean ± SD, none)	0.47 ± 0.190 (20 NAs)	0.52 ± 0.177 (0 NAs)	*p*-value: <0.001	*p*-value: <0.001 *p*-value: <0.001
Relaxation (mean ± SD, none)	0.34 ± 0.126 (20 NAs)	0.40 ± 0.154 (0 NAs)	*p*-value: 0.025	*p*-value: <0.001 *p*-value: 0.002
Interest (mean ± SD, none)	0.48 ± 0.093 (20 NAs)	0.51 ± 0.137 (0 NAs)	*p*-value: 0.139	*p*-value: <0.001 *p*-value: 0.017

## Data Availability

Data are unavailable due to privacy and ethical restrictions, but example scripts and a test environment can be found at https://github.com/SCT-lab/VRADHD [accessed on 1 February 2026].
